# In vivo mouse model of calcific myonecrosis induced by injury

**DOI:** 10.1371/journal.pone.0346816

**Published:** 2026-04-22

**Authors:** Mai Kuratani, Sho Tsukamoto, Ryo Hamai, Kaori Tsuchiya, Osamu Suzuki, Taketo Yamada, Takenobu Katagiri

**Affiliations:** 1 Division of Biomedical Sciences, Research Center for Genomic Medicine, Saitama Medical University, Saitama, Japan; 2 Division of Biomaterials Science and Engineering (Division of Craniofacial Function Engineering), Tohoku University Graduate School of Dentistry, Miyagi, Japan; 3 Department of Pathology, Saitama Medical University, Saitama, Japan; University of Texas Southwestern Medical Center, UNITED STATES OF AMERICA

## Abstract

Skeletal muscle is the major tissue that provides a space and source for bone, which is formed by osteoblasts through the secretion of extracellular matrices, such as type I collagen, and the deposition of hydroxyapatite calcium phosphate crystals. In the present study, we injected notexin, a snake venom, into skeletal muscle to induce local injury. Notexin injection caused local ectopic calcification in muscle fibers within a couple of days, which remained for more than 16 months. In contrast to ectopic bone formation, the damaged muscles after injection of notexin contains neither bone marrow cells, osteoblasts, nor osteoclasts. Histological and chemical analyses of the notexin-induced calcified objects revealed the deposition of stable hydroxyapatite crystals in muscle fibers, which was distinct from ectopic bone formation. We conclude that this in vivo mouse model of calcific myonecrosis induced by injury is useful for studying the molecular mechanisms of and potential therapeutics for calcific myonecrosis.

## Introduction

The bone matrix consists of organic and inorganic components, such as type I collagen and calcium phosphate crystals, respectively. Osteoblasts are unique cells responsible for bone formation; they secrete organic matrix components, called osteoids, as scaffolds to induce the deposition of inorganic mineral crystals. The prototype for bone apatite crystals is hydroxyapatite (Ca_10_(PO_4_)_6_(OH)_2_), which contains impurities such as carbonate and structural defects [1,2]. The apatite crystal phase is the most stable under physiological environments and is abundant among calcium phosphate crystals in the bone matrix. Octacalcium phosphate (OCP; Ca_8_H_2_(PO_4_)_6_•5H_2_O) has been suggested as a precursor of hydroxyapatite crystals in the early stage of bone formation [3–7]. The OCP/gelatin complex promotes angiogenesis and bone regeneration in a model of critical-sized bone defects of the rat calvarial artery [8]. Therefore, synthetic OCP is used as a scaffold in bone defects in the oral and maxillofacial regions [6,9–11]. The deposition of calcium phosphate crystals in the bone matrix is highly dependent on the enzyme activity of alkaline phosphatase (ALP), which is also expressed by osteoblasts on their cell membrane. In fact, loss-of-function mutations in nonspecific tissue ALP (the *ALPL* gene) cause immature bone tissue calcification, such as osteomalacia and rickets, in humans [12–14]. Bone-forming osteoblasts, which actively secrete components of the extracellular matrix, are histologically detected as single-lined cuboidal cells on the surface of the bone matrix. They are gradually embedded in the matrix secreted by themselves and become mature osteocytes.

Skeletal muscle is the major tissue that provides a location and source of bone formation in vertebrates. Transplantation of a growth factor called bone morphogenetic protein (BMP) in skeletal muscle induces a whole process of new bone formation through the endochondral ossification process [15–20]. Furthermore, the activation of BMP signaling by genetic gain-of-function mutations in a BMP receptor, *ACVR1*/ALK2, causes ectopic bone formation in skeletal muscles, tendons, and ligaments in patients with fibrodysplasia ossificans progressiva (FOP) [21–26]. BMP activity in target mesenchymal progenitor cells present in skeletal muscles induces the differentiation of cells into osteoblasts and chondrocytes. In skeletal muscle, ectopic bone due to new bone formation or calcification due to calcium build-up is induced by trauma, such as severe burns, explosions, and nerve injuries [27–30]. In fact, trauma to skeletal muscle has been reported to cause acute ectopic bone formation in patients with FOP and in a mouse model of FOP [31–36]. The inflammatory reactions induced by trauma may involve ectopic bone formation and calcification in skeletal muscle.

More than 60 years ago, Gallie and Thomson reported an unusual presentation of late myonecrosis with calcification that appeared decades after the trauma associated with Volkmann’s ischemic contracture [37]. This condition is known as calcific myonecrosis and is typically present in patients with injured lower extremities [38–41]. Furthermore, the development of calcific myonecrosis has been reported in patients after snake bite [42–44]. However, most clinical reports on trauma and calcification have been based on X-ray or CT scan analyses, and detailed pathological changes are still unclear.

A local injection of snake venom into skeletal muscle is used as a model of muscle regeneration induced by injury. In the present study, we injected notexin, a snake venom, into skeletal muscle to induce local injury. Notexin injection caused ectopic calcification in muscle fibers within a couple of days, which were maintained for more than 16 months. Histological and chemical analyses revealed the deposition of stable hydroxyapatite crystals in muscle fibers, which was distinct from the ectopic bone formation induced by BMP2. We concluded that this is an in vivo mouse model of injury-induced calcific myonecrosis and is useful for studying the molecular mechanisms of and potential therapeutics for calcific myonecrosis.

## Materials and methods

### Experimental animals

All animal experiments were approved by the Institutional Animal Care and Use Committee (IACUC) of Saitama Medical University (approval number: 3924) and were conducted in accordance with the animal experimentation regulations of Saitama Medical University. All the animal experiments were performed on 8-week-old male C57BL/6J mice purchased from CLEA Japan, Inc. (Tokyo, Japan). The mice were maintained on a 12-hour light/dark cycle and provided with unlimited food and water. The mice were randomly assigned to different experimental groups. Treatment and measurement order were randomized across experimental units to prevent systematic bias. No samples or animals were excluded from the analysis.

### Notexin‑induced muscle injury model

Following adequate anesthesia with isoflurane, 200 μl of 2 μg/ml notexin (Latoxan, Portes-Lès-Valence, France) dissolved in water was intramuscularly injected into the hind limb using a 1.0 ml 27 G insulin syringe. Specifically, 50 μl was injected into the tibialis anterior muscle and 150 μl into the gastrocnemius muscle. The whole muscles of the hind limb were analyzed by high-resolution μCT for the presence of hard tissue formation. Animals were sacrificed by isoflurane overdose, and hind limbs were collected at 1, 2, 3, 4 and 7 days after notexin injection. Day 7 samples were used to SEM and XRD analyses. The endpoint was defined as deterioration in general condition, including loss of appetite, difficulty urinating, abnormal posture, impaired locomotion, or self-injury. However, none of the mice met these criteria throughout the study period. The mice did not show any signs of diarrhea or distress during the notexin injection period.

### BMP-induced heterotopic ossification assay

Human BMP2 (Corefront Co., Tokyo, Japan) was transplanted into mice as pellets (4 mm diameter) with a collagen sponge as a carrier. Collagen pellets were generated via CollaTape (Zimmer Biomet Dental, Palm Beach Gardens, FL, USA) with a biopsy trepan. An incision on the skin and epimysium was made to expose the hamstring muscle in 8-week-old male C57BL/6J mice (CLEA Japan, Inc.), and a collagen pellet containing human BMP2 was implanted into the muscle tissue. Animals were sacrificed by isoflurane overdose, and hind limbs were collected at 2 weeks after implanted with a collagen pellet containing BMP2.

### High-resolution μCT

Hard tissue formation in the mice was scanned using CosmoScan GX (Rigaku, Yamanashi, Japan) with a field of view (FOV) of 25. The X-energy was set at 90 kV and 88 μA, and the exposure time was 4 min. 3D images were reconstructed from the μCT data obtained using CosmoScan GX software. Quantitative analysis of calcified muscle tissue was performed using CosmoScan GX and Analyze 12.0 software (AnalyzeDirect, Inc., Overland Park, KS, USA).

### Histological analysis

The hard skeletal muscle tissues were dissected and fixed with 4% paraformaldehyde (Nacalai Tesuque, Kyoto, Japan) in PBS at 4 °C overnight, embedded in paraffin, and used to prepare sections at a thickness of 4 μm using a Leica RM 2125RT rotary microtome (Leica Biosystems, Buffalo Grove, IL, USA). The sections were stained with hematoxylin-eosin, von Kossa and alizarin red S and analyzed with a BZ-9000 microscope (Keyence, Osaka, Japan). A TRAP & ALP double-staining kit purchased from TaKaRa Bio (Shiga, Japan) was used for the TRAP and ALP staining. The paraffin sections were deparaffinized with xylene. Endogenous peroxidase activity was quenched for 30 min with 0.3% H_2_O_2_. The sections were treated with blocking reagent (Nacalai Tesuque) at RT for 10 min and then sequentially incubated with primary antibodies against CD11b (rabbit monoclonal antibody; clone EPR1344 Abcam, Cambridge, UK), NLRP3 (rabbit polyclonal; Merck, Darmstadt, Germany), ASC (rabbit polyclonal; Merck), Caspase-1 (rabbit polyclonal; Abcam). The sections were incubated with an HRP-conjugated secondary antibody (Vector Laboratories, Burlingame, CA, USA) at RT for 30 min. Visualization was performed using an HRP substrate kit (Vector Laboratories), and the nuclei were counterstained with hematoxylin. For immunofluorescence staining, frozen tissue sections were used. After blocking with Blocking One Histo (Nacalai Tesque) for 10 min at room temperature (RT), the sections were incubated overnight at 4 °C with the following primary antibodies: anti-NLRP3 (rat monoclonal; R&D Systems, Minneapolis, MN, USA), anti-ASC (rabbit polyclonal; Merck), anti-caspase-1 (rabbit polyclonal; Abcam), laminin (rabbit polyclonal; Merck), myosin heavy chain type I (mouse monoclonal, clone BA-D5; DSHB, Iowa City, IA, USA), myosin heavy chain type IIa (mouse monoclonal, clone SC-71; DSHB), and myosin heavy chain type IIb (mouse monoclonal, clone BF-F3; DSHB). Alexa Fluor 488 plus-conjugated anti-rat IgG, Alexa Fluor 594 plus-conjugated anti-rabbit IgG, Alexa Fluor 647-conjugated anti-mouse IgG2b, Alexa Fluor 488-conjugated anti-mouse IgG1 and Alexa Fluor 555-conjugated anti-mouse IgM secondary antibodies (Thermo Fisher Scientific, Waltham, MA, USA) were applied for 1 hr at RT. Nuclei were counterstained with DAPI, and the sections were mounted using ProLong Gold Antifade Reagent (Thermo Fisher Scientific). Muscle fiber cross-sectional areas (CSAs) in frozen sections were quantified using a BZ-X800 microscope and BZ-Analyzer software (Keyence).

### Total RNA preparation and quantitative RT-PCR

After the whole gastrocnemius muscle was isolated, it was quickly frozen in liquid nitrogen, and a crushing machine (SK-200, Tokken, Kashiwa, Japan) was used to make the powder. The powder was treated with 1 ml of Sepasol RNA I Super G (Nacalai Tesuque) to remove proteins. Total RNA from tissues was prepared using a NucleoSpin RNA kit (Macherey-Nagel, Düren, Germany), and the RNA was reverse-transcribed to cDNA using cDNA EcoDry Premix (TaKaRa Bio). cDNA was used for the qPCR analysis with Premix Ex Taq (TaKaRa Bio). The qPCR analysis was performed using the TP800 qPCR System (TaKaRa Bio). The primers used are listed in S1 Table. The data are expressed as relative expression levels compared with those of *Gapdh*.

### Western blot analysis

The entire tibialis anterior muscle was isolated and homogenized in 20 volumes of Tris-HCl buffer (pH 7.4) using a Power Masher II homogenizer (Nippi, Tokyo, Japan). The homogenate was centrifuged at 13,000 g for 20 min at 4 °C, and the resulting supernatant was collected as the soluble protein fraction. Protein extracts were separated by sodium dodecyl sulfate-polyacrylamide gel electrophoresis (SDS-PAGE) and transferred onto polyvinylidene difluoride (PVDF) membranes as previously described. The membranes were incubated with primary antibodies against Runx2 (Cell Signaling Technology, Danvers, MA, USA) and α-tubulin (Cell Signaling Technology), followed by appropriate horseradish peroxidase-conjugated secondary antibodies. Protein bands were visualized using an enhanced chemiluminescence detection system (ChemiDoc XRS +, Bio-Rad Laboratories, Hercules, CA, USA).

### Enzyme-linked immunosorbent assay (ELISA)

Serum IL-18 concentrations were measured using the Mouse IL-18 DuoSet ELISA Kit (DY7625-05; R&D Systems) according to the manufacturer’s instructions. Blood was collected from the cardiac puncture, allowed to clot at room temperature for 30 min, and centrifuged at 1,500 g for 20 min to obtain serum. Serum samples were diluted 1:10 in Reagent Diluent and assayed. Absorbance was measured at 450 nm using a microplate reader. Concentrations were determined using a standard curve generated from recombinant mouse IL-18 standards.

### Scanning electron microscopy (SEM)

The hard skeletal muscle tissues were dissected and fixed with 4% paraformaldehyde in PBS at 4 °C overnight. The skeletal muscle tissue samples were stored in 99.5% ethanol before observation. The samples were dried at room temperature and then coated with Au-Pd. The microstructures of the samples were observed using a SEM (SEM, JSM-6390LA; JEOL LTD., Tokyo, Japan) at an acceleration voltage of 25 kV.

### X-ray diffraction (XRD)

The hard skeletal muscle tissues were dissected and fixed with 4% paraformaldehyde in PBS at 4 °C overnight. The skeletal muscle tissue samples were stored in 99.5% ethanol before observation. The samples were dried under the atmosphere at room temperature. The XRD patterns of the samples were measured using an X-ray diffractometer (Miniflex 600, Rigaku Co., Tokyo, Japan) with monochromatized Cu-Kα radiation (40 kV and 15 mA). The samples were scanned from 2θ = 3–60° with a scanning rate of 1.0°/min, and diffraction detection was applied at 0.02° step intervals. The International Centre for Diffraction Data (ICDD) was used to identify the phases of the crystals in the samples.

### Statistical analysis

Comparisons were made using unpaired one-way ANOVA and an unpaired t-test using GraphPad Prism 9 (GraphPad Software, San Diego, CA, USA). *P* values of < 0.05 were considered statistically significant.

## Results

### Notexin rapidly induces calcified objects in skeletal muscle fibers

**Fig 1:**
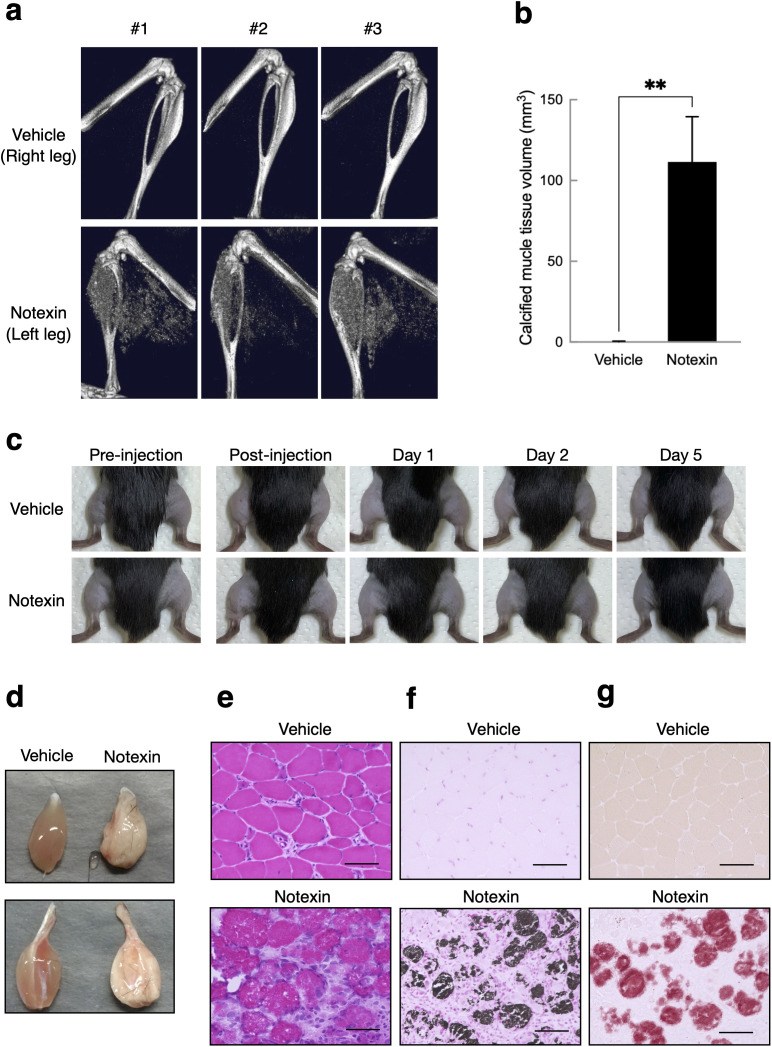
Snake venom, notexin, induces muscle injury and ectopic hard tissues in skeletal muscle. **(a)** 3D μCT reconstructions of the lower legs at 7 days after vehicle (left legs) or notexin (right legs) injection (n = 3). **(b)** Calcified tissue volume in the lower legs following vehicle or notexin injection (n = 3), as determined by µCT analysis. Data are presented as mean ± S.D. Statistical significance was assessed using unpaired t-test (**P < 0.01). **(c)** Gross morphology of hind limbs before and after notexin injection. The hair on both lower legs was removed, and notexin was injected into the left legs. **(d)** Tibialis anterior muscles (upper panel) and skeletal muscles, which include the gastrocnemius, plantaris and soleus muscles (lower panel), were prepared from mice at 7 days after injection with vehicle or notexin. **(e)** Paraffin sections of the gastrocnemius muscle in mice injected with 0.4 μg of notexin or vehicle. The sections were stained with hematoxylin & eosin. The scale bars represent 100 μm. **(f, g)** Paraffin sections of gastrocnemius muscle prepared from mice injected with vehicle or notexin were stained with von Kossa stain **(f)** and alizarin red S stain **(g)**. The scale bars represent 100 μm.

We injected 0.4 μg of notexin into the hindlimb skeletal muscles of wild-type C57BL/6J male mice to induce muscle injury and regeneration in vivo. On Day 7 after injection, opaque ectopic radiograph images were obtained via μCT analysis in all notexin-injected skeletal muscles but not in the vehicle-injected skeletal muscles (Fig 1a and 1b). No significant edema was observed in the left hind limbs of mice injected with either notexin or vehicle (Fig 1c). Notexin-injected mice showed no significant differences in body weight changes compared with control mice throughout the experimental period (S1a Fig). Furthermore, at Day 19, skeletal muscles were harvested, body weight-normalized muscle weights did not differ significantly between the two groups (S1b and c Fig). In contrast, at Day 7 post-notexin injection, tibialis anterior (TA) and gastrocnemius muscles were harvested and morphologically normalized by tibia length, revealing significantly reduced muscle weights in both muscles compared to controls (S2a and b Fig). Laminin immunostaining of TA muscle sections, followed by quantification of myofiber cross-sectional area (CSA), revealed significantly reduced fiber sizes in notexin-injected muscles compared with controls (S2c and d Fig). Myosin heavy chain (MyHC) immunostaining demonstrated that calcified myofibers in notexin-injected muscles were negative for MyHC isoforms, including type I, type IIa, and type IIb (S3 Fig). Compared with those from the vehicle-injected skeletal muscles, the skeletal muscles from the mice injected with notexin were swollen and whitish (Fig 1d). Hematoxylin-eosin staining of sections prepared from skeletal muscles injected with and without notexin revealed that notexin induced an infiltration of mononuclear cells into the interstitial area around each muscle fiber (Fig 1e). Both alizarin red S and von Kossa, which detect calcium and phosphate, respectively, stained the damaged muscle fibers but not the interstitial area infiltrated by the mononuclear cells in the notexin-injected skeletal muscles (Fig 1f and 1g). These results suggest that the objects in the skeletal muscle fibers induced by notexin injection contained calcium phosphate crystals.

**Fig 2:**
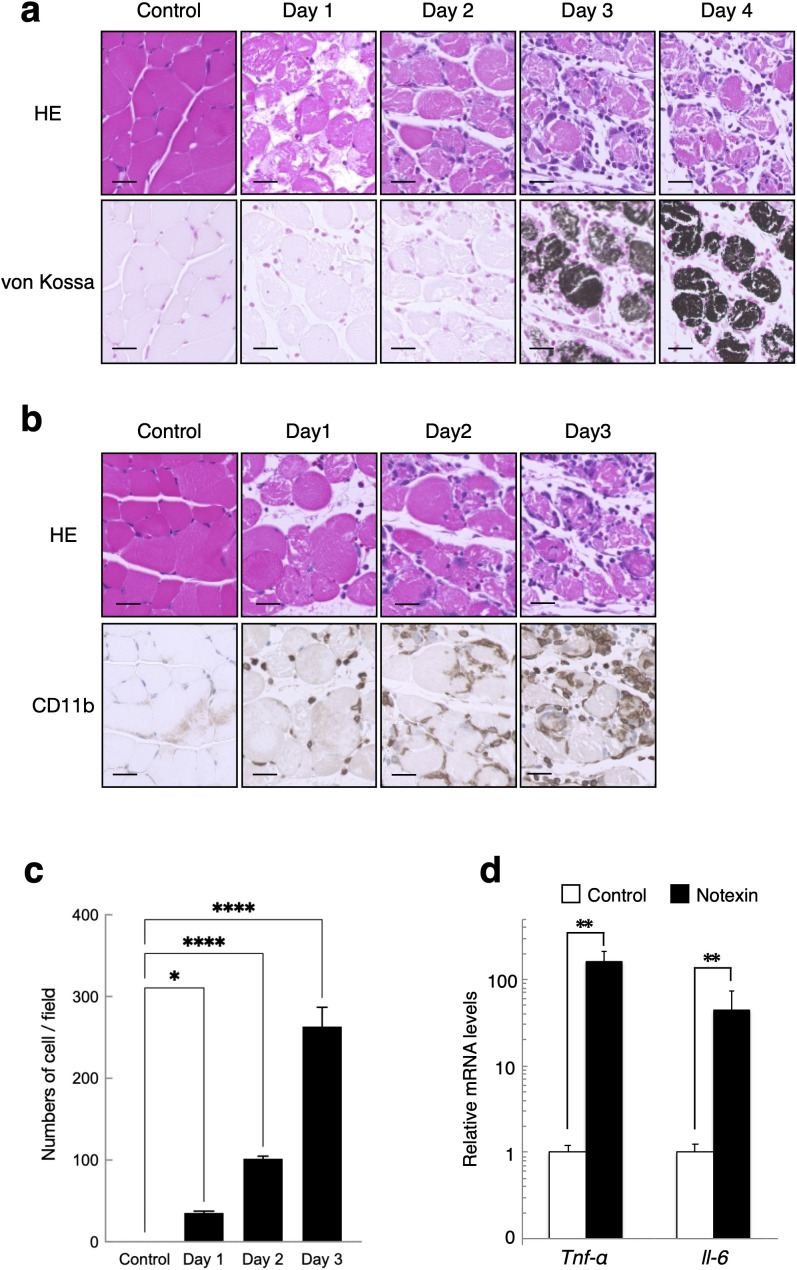
Calcified muscle fibers were detected after muscle inflammation. **(a)** Time-course changes in the gastrocnemius muscle at 1 day, 2 days, 3 days and 4 days after injection with notexin and uninjured muscle (control). The sections were subjected to hematoxylin & eosin (upper panel) and von Kossa staining (lower panel) (n = 3). The scale bars represent 50 μm. **(b)** Sections of the gastrocnemius muscle at 1 day, 2 days and 3 days after injection with notexin were immunostained with an anti-CD11b antibody. The scale bars represent 50 μm. **(c)** The number of CD11b-positive cells per field was counted in randomly selected fields at ×40 magnification. Data are presented as mean ± S.D. (n = 3 fields per group). **(d)** Quantitative RT-PCR analysis of *Tnf-a*and *Il-6* genes in skeletal muscles in control and notexin injected mice (n = 3). Data are expressed as mean ± S.D. P values are calculated using unpaired one-way ANOVA (**c**) or unpaired t-test **(d)** (*P < 0.05, **P < 0.01, ****P < 0.0001).

We analyzed the earlier time course changes in the skeletal muscle fibers injected with notexin. Notexin injection induced von Kossa-stained muscle fibers between Day 2 and Day 3 (Fig 2a). Immunohistochemical staining with an antibody against CD11b, which is a surface marker of inflammatory cells, such as monocytes, macrophages, and granulocytes, revealed that those inflammatory cells infiltrated the interstitial area in response to notexin injection on Day 1 (Fig 2b and 2c). The number of CD11b-positive cells gradually increased until Day 3, when the muscle fibers appeared to be stained with von Kossa (Fig 2a and 2b). The expression levels of *TNF-α* and *IL-6* mRNAs quantified by RT-qPCR were increased by notexin injection on Day 3, suggesting that notexin injection induced inflammation in skeletal muscles (Fig 2d).

### Notexin promotes muscle calcification via inflammasome activation

**Fig 3:**
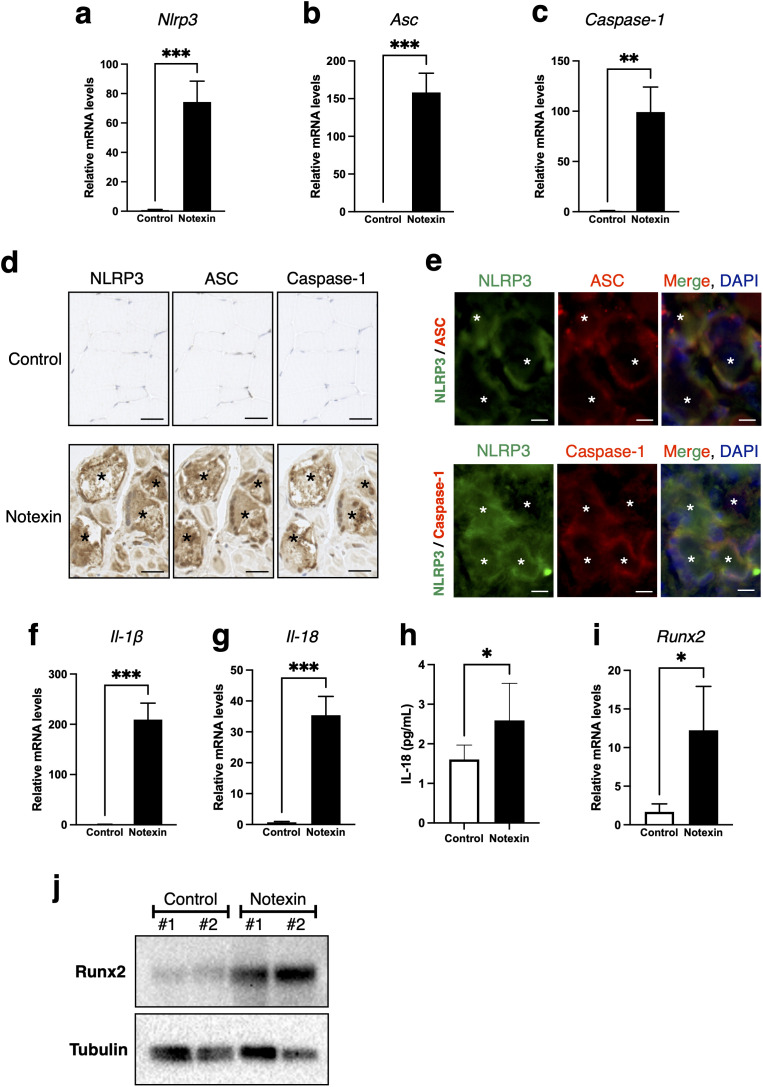
Notexin promotes skeletal muscle calcification via NLRP3 inflammasome activation and Runx2 upregulation. **(a-c)** Quantitative RT-PCR analysis of *Nlrp3* **(a)**, *Asc* **(b)**, and *Caspase-1* **(c)** mRNA expression in skeletal muscle from control and notexin-injected mice (n = 3). Data are presented as mean ± S.D. Statistical significance was assessed using unpaired t-test (**P < 0.01, ***P < 0.001). **(d)** Immunostaining of gastrocnemius muscle sections 3 days after notexin injection using anti-NLRP3, anti-ASC, and anti-Caspase-1 antibodies on serial sections. The upper panels show control muscles, and the lower panels show notexin-treated muscles. Scale bars, 20 μm. Asterisks indicate calcified muscle fibers. **(e)** Immunofluorescence staining showing the localization of NLRP3 (green), ASC or Caspase-1 (red) around calcified muscle fibers in notexin-injected skeletal muscle. Nuclei are counterstained with DAPI (blue). Scale bar, 20 μm. Asterisks indicate calcified muscle fibers. **(f, g)** Quantitative RT-PCR analysis of *Il-1β* **(f)** and *Il-18* **(g)** mRNA expression in skeletal muscle from control and notexin-injected mice (n = 3). Data are presented as mean ± S.D. Statistical significance was assessed using unpaired t-test (***P < 0.001). **(h)** Serum IL-18 levels measured by ELISA in control and notexin-injected mice (n = 6 (control), n = 9 (notexin)). Data are presented as mean ± S.D. Statistical significance was assessed using unpaired t-test (*P < 0.05). **(i)** Quantitative RT-PCR analysis of *Runx2* mRNA expression in skeletal muscle from control and notexin-injected mice (n = 3). Data are presented as mean ± S.D. Statistical significance was assessed using unpaired t-test (*P < 0.05). **(j)** Western blot analysis of Runx2 and tubulin (loading control) protein expression in skeletal muscle from control and notexin-injected mice.

We investigated whether notexin administration activates the inflammasome pathway and promotes calcification in skeletal muscle. Muscles injected with notexin exhibited increased mRNA expression of *Nlrp3, Asc*, and *Caspase-1* compared with controls (Fig 3a, 3b and 3c). Immunohistochemical analysis of serial sections from control and notexin-injected muscles revealed that these proteins were specifically localized within the calcified muscle fibers in notexin-injected mice (Fig 3d). Immunofluorescence analysis further demonstrated co-localization of NLRP3 with ASC and Caspase-1, indicating activation of the inflammasome pathway in notexin-injected muscle fibers (Fig 3e). The mRNA levels of *Il-1β* and *Il-18*, downstream cytokines of the inflammasome pathway, were also elevated in notexin-injected muscles compared with controls (Fig 3f and 3g). Consistently, serum IL-18 levels were significantly higher in the notexin group as determined by ELISA (Fig 3h). In addition, both mRNA and protein expression of Runx2, a transcription factor associated with calcification, were significantly upregulated in notexin-injected muscles (Fig 3i and 3j). Collectively, these findings indicate that notexin promotes skeletal muscle calcification through the NLRP3 inflammasome and induction of Runx2.

### Notexin-induced calcification involves the formation of hydroxyapatite crystals, which are stable in skeletal muscles

**Fig 4:**
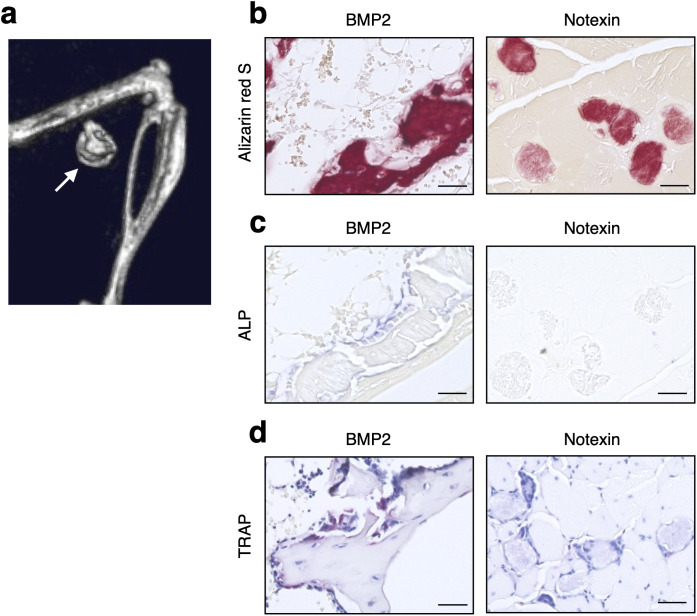
Notexin injection-induced hard tissues in skeletal muscle are calcified rather than ossified. **(a)** Mice were implanted with a collagen pellet containing 2 μg of BMP2 in the hamstring muscle. μCT images of the mice were obtained at 14 days after implantation. The arrow indicates heterotopic ossification of the skeletal muscle. **(b-d)** Sections of skeletal muscles from mice injected with notexin or implanted with 2 μg of BMP2 (heterotopic ossification). The sections were subjected to alizarin red S staining **(b)**, alkaline phosphatase staining **(c)** and TRAP staining **(d)**. The scale bars represent 50 μm.

We implanted 2 μg of BMP2 into the skeletal muscle of wild-type C57BL/6J male mice to induce ectopic bone formation after 2 weeks (Fig 4a). Nondemineralized sections of ectopic bone induced by BMP2 were stained with alizarin red S, similar to the muscle fibers injected with notexin (Fig 4b). Cuboidal osteoblasts identified by alkaline phosphatase (ALP) activity and osteoclasts identified by tartrate-resistant acid phosphatase (TRAP) activity were observed on the bone matrix induced by BMP2 in the skeletal muscle (Fig 4c and 4d). In contrast, neither osteoblasts nor osteoclasts were observed in the muscle fibers injected with notexin, even though they were stained with alizarin red S (Fig 4b, 4c and 4d).

**Fig 5:**
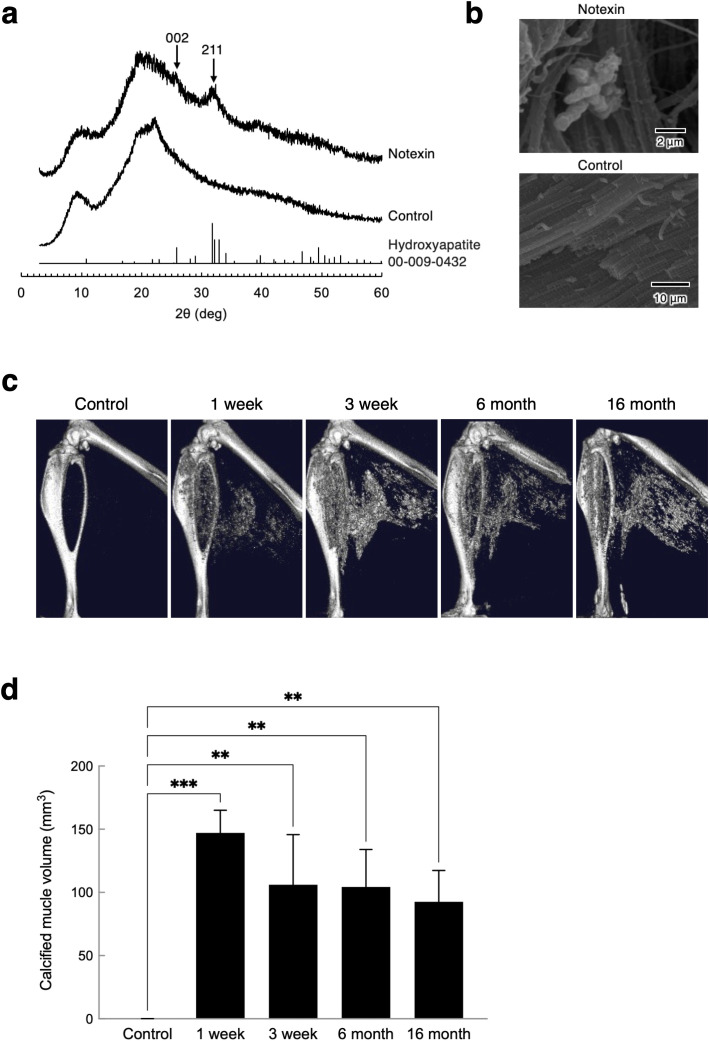
Notexin injection-induced hard tissues in skeletal muscle are calcified rather than ossified. **(a)** X-ray diffraction (XRD) patterns of the gastrocnemius and hamstrings of the control (uninjured) and notexin-injected groups on Day 7. The arrows indicate the broad diffractions attributed to the (002) and (211) planes of hydroxyapatite (ICDD, 00-009-0432). **(b)** Scanning electron microscopy (SEM) images of control (uninjured) or notexin-injected muscle. The scale bars represent 2 μm (upper panel) and 10 μm (lower panel). **(c)** 3D μCT reconstructions of long-term changes in the lower legs of the same mouse at 1 week, 3 weeks, 6 months and 16 months after injection with notexin. **(d)** Long-term changes in calcified volume were quantitatively analyzed (n = 3). Data are expressed as mean ± S.D. *P* values are calculated using unpaired one-way ANOVA (**P < 0.01, ***P < 0.001).

To identify the crystal phases of the deposits formed by notexin in the fibers of the skeletal muscle, we analyzed them by X-ray diffraction (XRD). Broad peaks were observed at approximately 2θ = 9 and 20° in the XRD patterns of the skeletal muscles with and without notexin injection. However, broad diffractions attributed to (002) and (211) of hydroxyapatite (ICDD, 00-009-0432) were detected at 2θ = 25.9 and 31.7°, respectively, in the pattern of the sample prepared from the skeletal muscles injected with notexin (Fig 5a). The XRD patterns indicated that low-crystalline hydroxyapatite was formed by notexin in the skeletal muscles. In the scanning electron microscopy (SEM) images, rod-shaped crystals were observed in the skeletal muscles injected with notexin but not in the control muscle (Fig 5b). We investigated the calcified objects induced by notexin in the skeletal muscles by μCT analysis and found that they remained for 16 months in vivo after notexin injection (Fig 5c and 5d).

## Discussion

Calcified tissues are ectopically formed in skeletal muscle after injuries secondary to traffic accidents and burns [27–30]. In the present study, we established a mouse model of ectopic calcification in skeletal muscles by injecting snake venom, notexin, into wild-type C57BL/6J mice. We found that notexin injection induced calcification, which involves calcium and phosphate, in the fibers of the skeletal muscle within several days. The induced calcified objects were stable and remained in the skeletal muscles for more than 1 year in vivo. Octacalcium phosphate (OCP), a form of physiological calcium phosphate crystal, is detected early in the formation of normal bone in vivo [6,7]. In vivo OCP transplants are resorbable and gradually replaced with normal bone tissue by osteoclasts and osteoblasts [10,45]. Therefore, OCPs are expected to be useful crystals for the replacement of bones with artificial ones in the orthopedic field [6,10,11,45]. The notexin-induced calcified objects in the skeletal muscles consisted of hydroxyapatite crystals similar to mature bone. However, the notexin-induced calcified objects did not contain bone cells, such as osteoblasts, osteoclasts, or bone marrow cells. Osteoblasts in the ectopic bones of the skeletal muscles induced by BMP2 were differentiated from mesenchymal progenitor cells, which are positive for the surface markers PDGFRα, Sca-1, and Tie-2 but not myogenic lineage cells [46–48]. The injection of notexin induced calcification in the muscle fibers but not in the surrounding interstitial area, where there is space for mesenchymal progenitor cells to grow and differentiate during ectopic bone formation in skeletal muscles [46,49]. Patients with calcific myonecrosis in the legs exhibit extensive calcification even decades after tibial fracture [38]. In the present study, we also detected calcified objects more than 16 months after notexin injection into the skeletal muscles of the mice. These findings indicate that there are two different types of calcification in skeletal muscles: ectopic bone formation and crystal deposition. The latter case involves calcification induced by muscle injury from notexin injection in mice and traffic accidents in humans. To our knowledge, no mouse model specifically representing myonecrosis has previously been reported. Therefore, the model established in this study is the first to mimic myonecrosis. This novel model provides a valuable tool for investigating the pathophysiology of myonecrosis. Moreover, it is useful for studying ectopic calcification caused by myonecrosis in skeletal muscle, enabling the examination of the molecular mechanisms underlying calcification as well as the evaluation of potential therapeutics to prevent and/or eliminate crystal deposition in vivo. Nonetheless, it should be noted that this model does not fully replicate all pathological features of human calcific myonecrosis.

Calcium ions are the most important minerals for muscle contraction and are stored in the sarcoplasmic reticulum of skeletal muscles. In the notexin-injected muscles, calcification was observed in the muscle fibers but not in the interstitial area. It is possible that the ionized calcium stored in muscle can form hydroxyapatite crystals with phosphorus in muscle fibers as a scaffold for muscle damage. This hypothesis is supported by a report of diaphragm calcification in *mdx* mice, which is a pathological model of human Duchenne muscle dystrophy [50]. In genetic muscular dystrophies characterized by defects in the sarcolemma, increased calcium influx results in mitochondrial calcium overload. This cascade precipitates mitochondrial dysfunction, impaired energy metabolism, and defective membrane repair, thereby exacerbating muscle fiber damage and degeneration. Consequently, mitochondrial dysfunction emerges as a pivotal factor in the progression of disease and muscle fiber necrosis [51,52]. Recent studies have further elucidated the mechanism by which mitochondria contribute to this pathology. In particular, the mitochondria-associated membranes (MAMs) serve as critical hubs for calcium transfer from the sarcoplasmic reticulum to mitochondria, mediated by key proteins such as GRP75 and VDAC1 [53,54]. Modulation of these proteins has been shown to reduce mitochondrial calcium overload, thereby alleviating mitochondrial dysfunction and significantly decreasing pathological calcium deposition in muscle tissue. Dubinin et al. demonstrated that inhibition of GRP75 effectively reduces mitochondrial calcium overload and muscle calcification in dystrophic models [54]. Furthermore, the calcium implicated in ectopic calcification may also originated from the extracellular space, particularly following disruption of the basal lamina of muscle fibers. Detailed ultrastructural analyses are warranted to elucidate the precise source of calcium responsible for driving ectopic calcification. Notexin is a presynaptic phospholipase A2 (PLA2) derived from tiger snake venom that inhibits the release of acetylcholine in the presynaptic membrane and has strong muscle toxicity [55–57]. Consistent with previous studies, injection of notexin in our experimental model also resulted in apparent muscle atrophy [57]. These results support the formation of calcified muscle fibers during the muscle regeneration processes. Hydrolysis of the cell membrane leads to breakdown of the internal structure of muscle fibers [55–57]. In the present study, notexin did not destroy the rough structures of muscle fibers, including the basal lamina, as previously reported [58,59]. We confirmed that notexin led to the infiltration of inflammatory cells and increased the expression of inflammatory cytokines, such as *TNF-α* and *IL-6*. It might be possible that notexin-induced inflammation enhances the deposition of hydroxyapatite crystals in the muscle fibers by disrupting the membrane structures through proteases secreted by inflammatory cells. In addition, inflammatory cells may secrete components that serve as the scaffolds for crystal deposition. Additionally, we demonstrated that notexin activates the NLRP3 inflammasome pathway in skeletal muscle, as evidenced by upregulated mRNA expression of *Nlrp3*, *Asc*, and *Caspase-1* with protein localization around calcified fibers. Inflammasome activation increased local and systemic IL-1β/IL-18 production alongside Runx2 expression at mRNA and protein levels. This molecular cascade mirrors mechanisms in calcific aortic valve disease (CAVD), where NLRP3 inflammasome activation drives IL-1β/IL-18 elevation and Runx2 induction to promote valvular calcification [60,61]. The synergism of inflammation and calcium ions induces rapid deposition of hydroxyapatite crystals on necrotic muscle fibers by notexin injection. However, the detailed molecular cascade linking membrane damage, calcium release from the sarcoplasmic reticulum, and the nucleation of hydroxyapatite crystals remains to be elucidated. In future studies, we plan to investigate calcium dynamics, the expression of related molecules, and perform ultrastructural analyses to clarify the underlying mechanisms in greater detail. Additional studies are needed to elucidate the molecular mechanisms that underlie this pathological event in vivo.

## Supporting information

S1 Fig Body weight remain unchanged after notexin injection. (a) Time-course of body weight changes in control and notexin-injected mice (n = 5). Data are presented as mean ± SD. (b and c) Wet weights of the tibialis anterior (b) and gastrocnemius muscles (c) from control and notexin-injected mice at 19 days post-injection (n = 5). Data are presented as mean ± S.D.
(TIFF)

S2 Fig Notexin-induced muscle atrophy. (a and b) Tibia length-normalized wet weights of the tibialis anterior (TA) (a) and gastrocnemius muscles (b) from control and notexin-injected mice at 7 days post-injection (n = 5). Data are presented as mean ± S.D. Statistical significance was assessed using unpaired t-test (**P < 0.01). (c) Representative images of laminin immunostaining in TA muscle cryosections from control and notexin-treated mice at Day 7 post-injection. Scale bars represent 100 μm. (d) Quantification of myofiber cross-sectional area (CSA) from laminin-stained TA sections (n = 3000–5500 fibers/muscle, 3 mice/group). Data are mean ± SD.
(TIFF)

S3 Fig Muscle fiber type analysis of notexin-induced calcified myofibers. MyHC immunostaining of TA muscle cryosections from control and notexin-treated mice at Day 19 post-injection. Representative images show MyHC-type I (bright blue), type IIa (green), and type IIb (red). Scale bars, 20 μm. Insets highlight calcified regions containing MyHC-negative myofibers (asterisks).
(TIFF)

S1 Table Primer sequences used for quantitative real-time PCR analysis of inflammasome- and calcification-related genes. (TIFF)

S2 Table Data quality summary. (XLSX)
