# Supplementary figures and images for: In vivo mouse model of calcific myonecrosis induced by injury
Source: PLoS One. 2026 Apr 22;21(4):e0346816. doi: 10.1371/journal.pone.0346816 (PMC13102222; doi:10.1371/journal.pone.0346816)

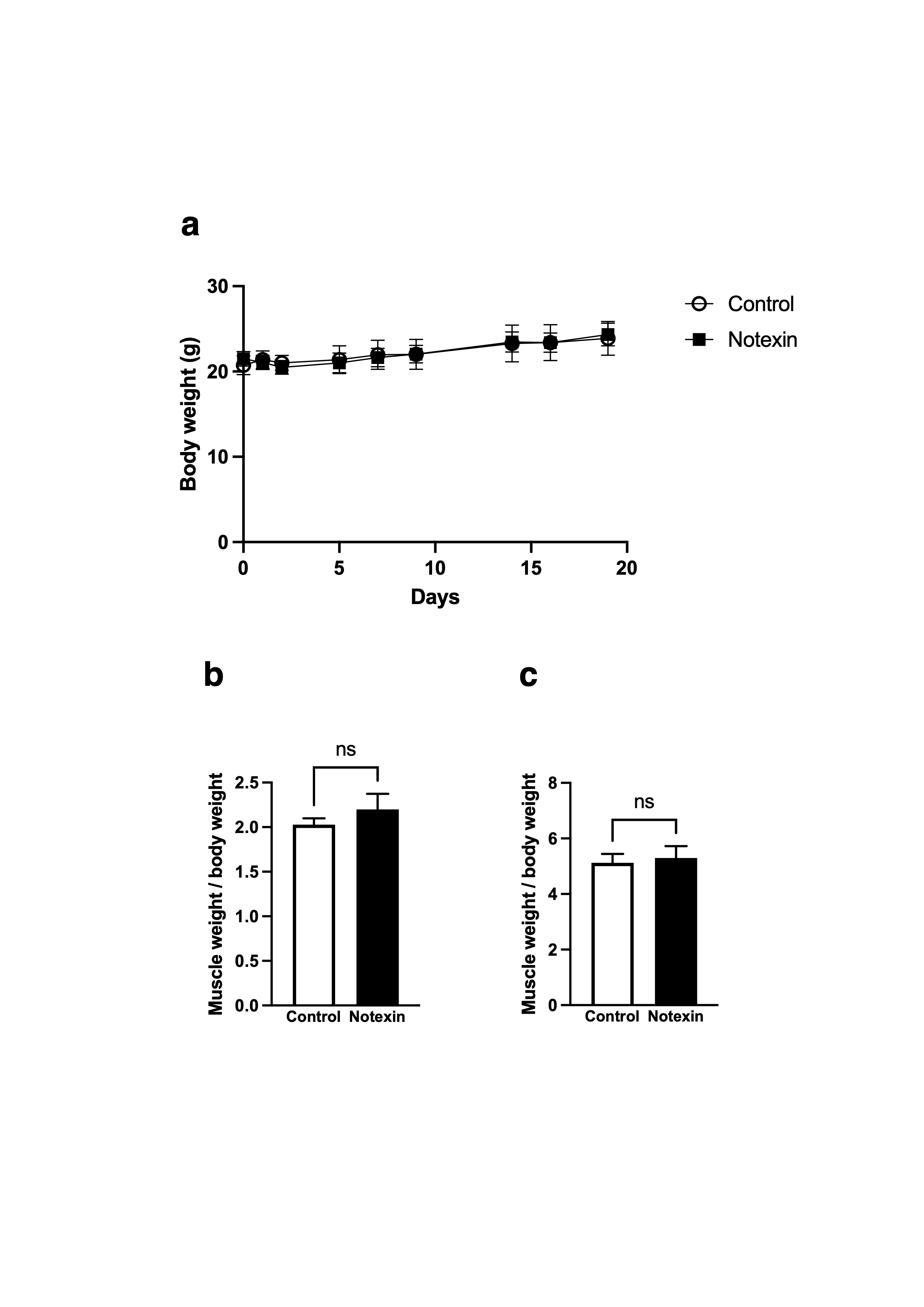

Supplement: S1 Fig — (a) Time-course of body weight changes in control and notexin-injected mice (n = 5). Data are presented as mean ± SD. (b and c) Wet weights of the tibialis anterior (b) and gastrocnemius muscles (c) from control and notexin-injected mice at 19 days post-injection (n = 5). Data are presented as mean ± S.D. (TIFF) [file pone.0346816.s001.tiff]

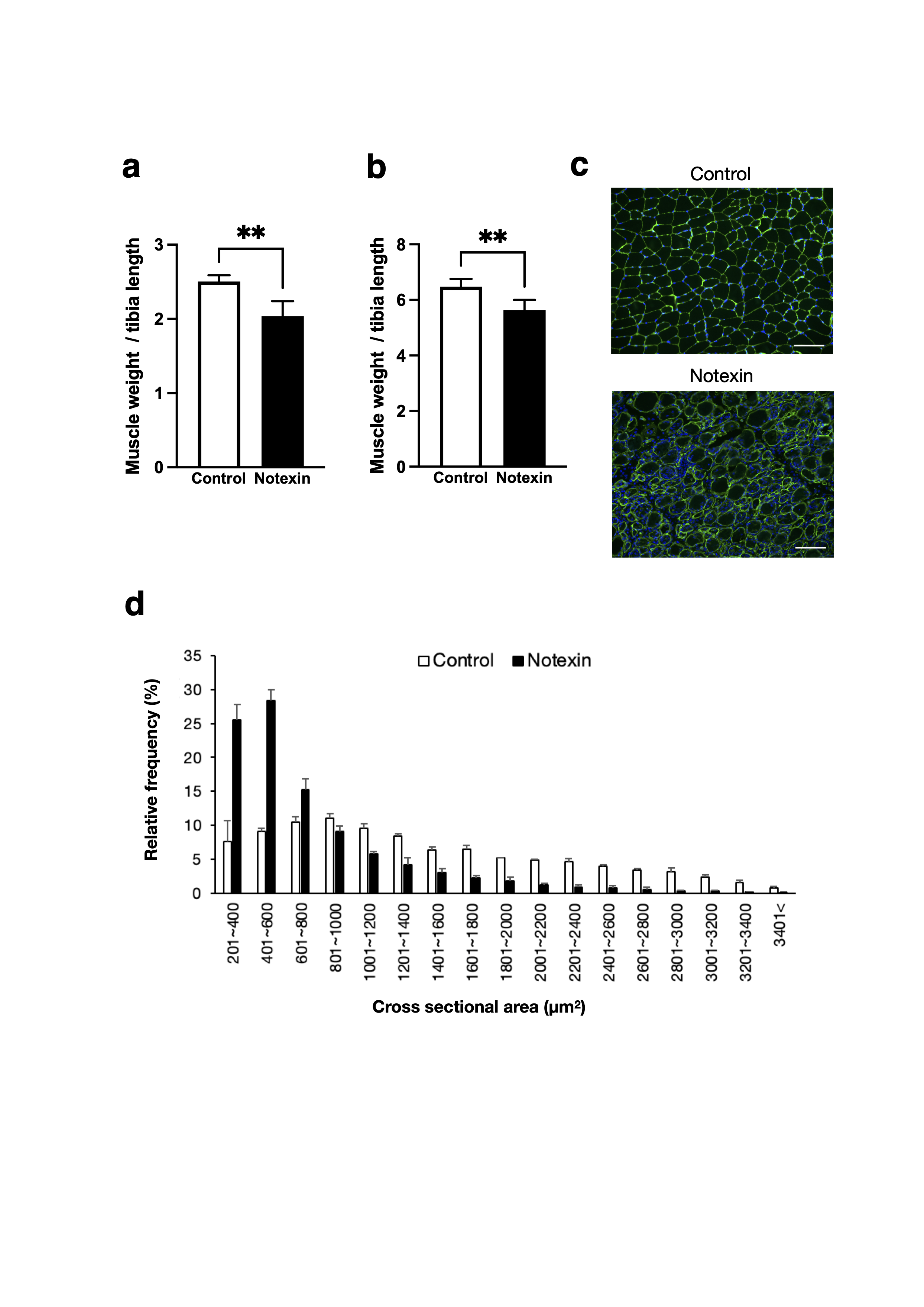

Supplement: S2 Fig — (a and b) Tibia length-normalized wet weights of the tibialis anterior (TA) (a) and gastrocnemius muscles (b) from control and notexin-injected mice at 7 days post-injection (n = 5). Data are presented as mean ± S.D. Statistical significance was assessed using unpaired t-test (**P < 0.01). (c) Representative images of laminin immunostaining in TA muscle cryosections from control and notexin-treated mice at Day 7 post-injection. Scale bars represent 100 μm. (d) Quantification of myofiber cross-sectional area (CSA) from laminin-stained TA sections (n = 3000–5500 fibers/muscle, 3 mice/group). Data are mean ± SD. (TIFF) [file pone.0346816.s002.tiff]

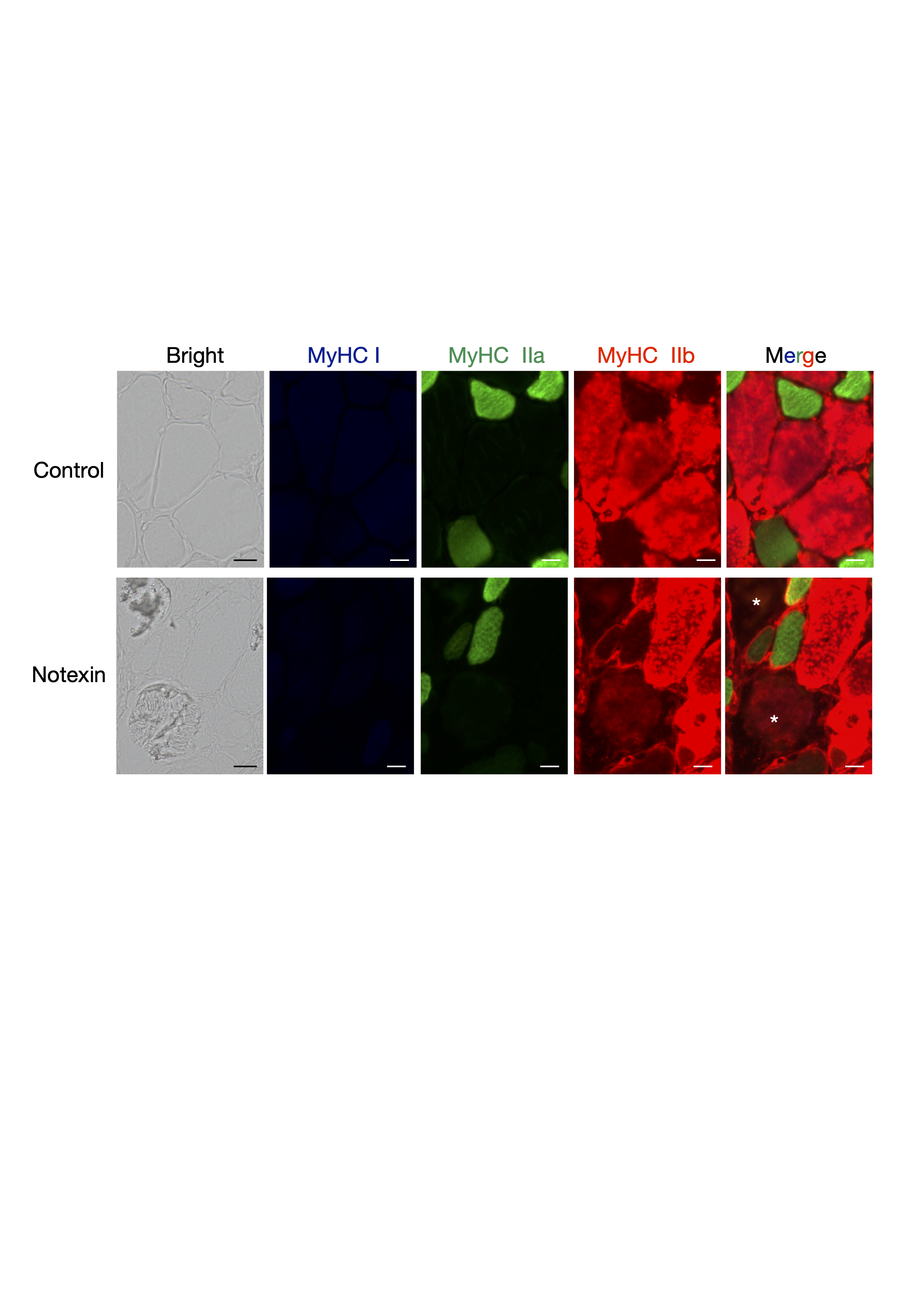

Supplement: S3 Fig — MyHC immunostaining of TA muscle cryosections from control and notexin-treated mice at Day 19 post-injection. Representative images show MyHC-type I (bright blue), type IIa (green), and type IIb (red). Scale bars, 20 μm. Insets highlight calcified regions containing MyHC-negative myofibers (asterisks). (TIFF) [file pone.0346816.s003.tiff]

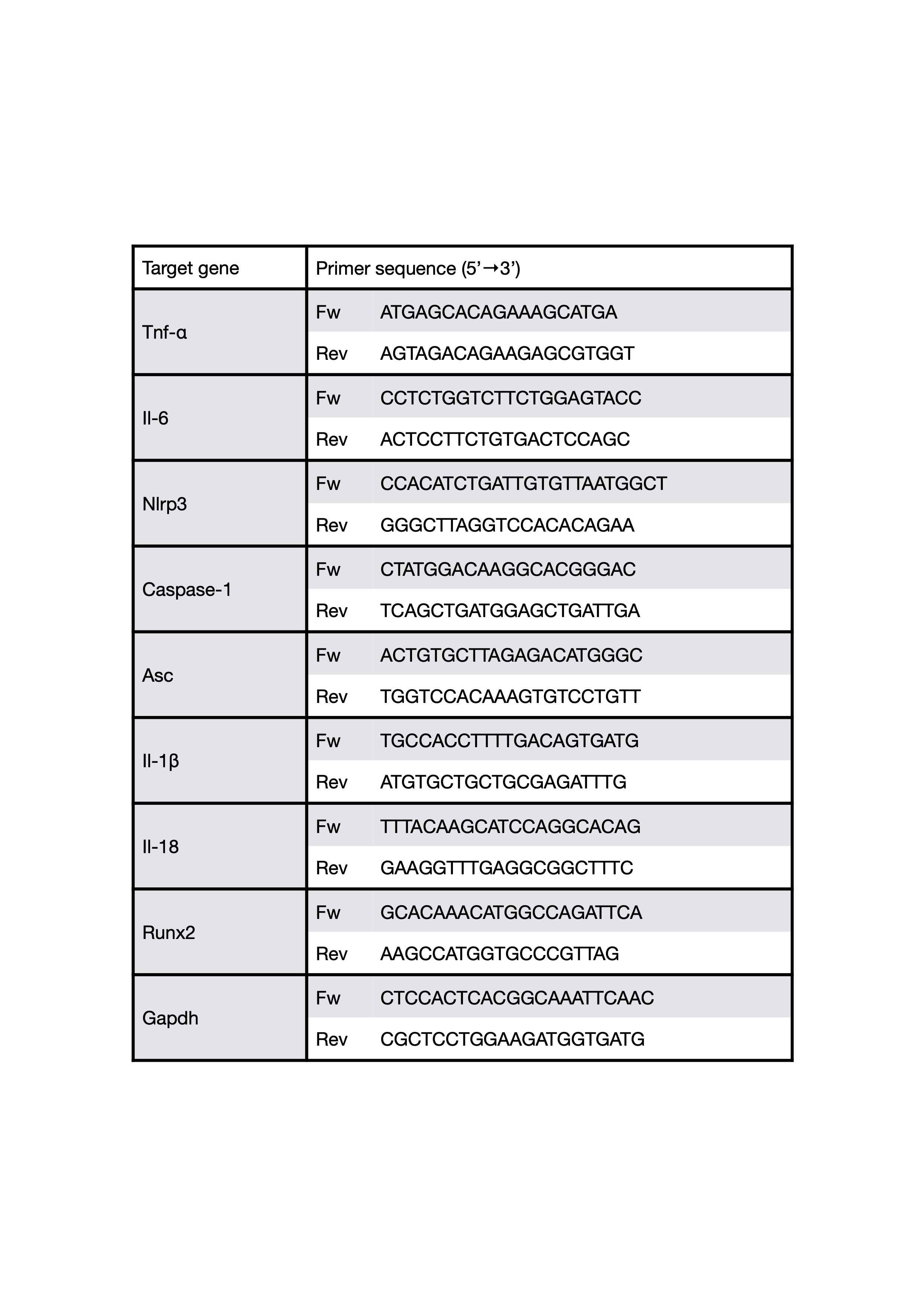

Supplement: S1 Table — (TIFF) [file pone.0346816.s004.tiff]
